# Quantitative proteomics identifies clusterin as a novel biomarker for atherosclerosis

**DOI:** 10.1002/ame2.70143

**Published:** 2026-02-02

**Authors:** Dengfeng Ding, Yingjie Zhang, Li Zhang, Xinou Zheng, Miaomiao Niu, Yunxiao Jia, Xuezhuang Li, Hua Chen, Chao Guo, Tao Jiang, Yuqiong Zhao

**Affiliations:** ^1^ Medical Innovation Research Department Chinese PLA General Hospital Beijing China; ^2^ Department of Nephrology First Medical Center of Chinese PLA General Hospital, Nephrology Institute of the Chinese People's Liberation Army, State Key Laboratory of Kidney Diseases Beijing China; ^3^ Beijing Engineering Research Center for Experimental Animal Models of Human Diseases Peking Union Medicine College Beijing China

**Keywords:** AKT, atherosclerosis, clusterin, LRP1, proteomics

## Abstract

**Background:**

Atherosclerosis (AS), the leading cause of cardiovascular disease, involves complex molecular mechanisms that remain incompletely understood, particularly in the context of diet‐induced vascular lesions.

**Methods:**

We established an AS model in Bama miniature pigs using a high‐cholesterol, high‐fat diet (HCFD) and performed quantitative proteomic analysis on coronary artery tissues. Key proteins were identified using protein–protein interaction (PPI) network analysis and subsequently validated by histopathological evaluation in porcine and murine coronary arteries. The underlying molecular mechanisms were elucidated using Western blot analysis.

**Results:**

The HCFD successfully induced an atherosclerotic phenotype characterized by significantly elevated serum lipid levels. Proteomic analysis identified 108 differentially expressed proteins (DEPs) between the AS and control groups. From four identified hub proteins, we focused on clusterin (CLU), which was markedly upregulated in atherosclerotic coronary tissues, particularly within endothelial cells (ECs) and smooth muscle cells (SMCs). Mechanistically, CLU upregulation activated the LRP1/AKT signaling pathway, thereby promoting atherogenesis.

**Conclusion:**

Our study reveals that elevated CLU expression accelerates the process of AS by activating the LRP1/AKT pathway. These data elucidate a novel pro‐atherogenic role for CLU and establish the CLU/LRP1/AKT axis as a promising therapeutic target for managing AS, particularly in pathologies driven by high‐fat diets.

## Graphical abstract

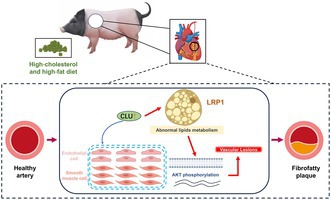

## 1 INTRODUCTION

Atherosclerosis (AS), a chronic inflammatory disease, is the principal underlying cause of most cardiovascular events, which often result from the progression or rupture of atherosclerotic plaques.^1^ Endothelial cells (ECs) and smooth muscle cells (SMCs) are known to play pivotal roles throughout all stages of this pathology.^2^, ^3^

To effectively model the human condition, the choice of an appropriate animal model is critical. The miniature pig is considered an excellent model for AS research due to its profound resemblance to humans in cardiovascular physiology, lipoprotein metabolism, and the morphology of pathological plaques.^4^, ^5^, ^6^ Notably, a high‐cholesterol, high‐fat diet (HCFD) can effectively accelerate the progression of AS in this model, mirroring diet‐induced pathology in humans.^4^, ^7^

The currently commonly used biomarkers for AS are primarily divided into two major categories: lipid metabolism‐related and inflammation‐related, and the most representative biomarkers are low‐density lipoprotein cholesterol (LDL‐C) and C‐reactive protein (CRP), respectively.^8^, ^9^ Although they are widely used in clinical risk assessment, both have certain limitations. LDL‐C only reflects the total amount of cholesterol carried by LDL particles and cannot distinguish the number, size, or subtypes of particles, making it difficult to accurately assess risks.^10^ CRP lacks disease specificity and can be significantly elevated in various non‐AS conditions such as infections, trauma, autoimmune diseases, and malignancies. Its predictive value is substantially diminished when other vascular risk factors are incorporated.^11^ Additionally, some patients with early‐stage or mild AS may not exhibit significant increases in LDL‐C and CRP levels, potentially leading to missed diagnoses.^10^, ^11^ Clusterin (CLU), also referred to as apolipoprotein J (ApoJ), is a multifunctional chaperone protein exhibiting diverse and often contradictory biological roles.^12^, ^13^ Although CLU is recognized for its neuroprotective functions in Alzheimer's disease and its pro‐survival role in cancer, its contribution to cardiovascular disease is only beginning to be understood.^14^, ^15^, ^16^ Recent studies suggest CLU may be a therapeutic target, as its involvement in early stages of key processes such as lipid transport and SMC differentiation can be detected.^12^, ^17^, ^18^, ^19^ However, the specific mechanisms through which CLU influences AS remain largely unknown.

In this study, we aimed to elucidate the role and underlying mechanisms of CLU in a porcine model of advanced, diet‐induced AS. We hypothesized that CLU expression is elevated in atherosclerotic lesions, and that it contributes to disease progression by modulating key signaling pathways within vascular ECs and SMCs. By investigating this hypothesis, we aim to uncover a novel molecular axis in the pathology of AS and provide a potential novel target for therapeutic intervention.

## 2 METHODS

### 2.1 Porcine model of atherosclerosis

All animal procedures received approval from the Institutional Animal Care and Use Committee (IACUC) of the Chinese PLA General Hospital (approval ID: 2018‐D14‐26) and were conducted in accordance with their guidelines. A total of 12 male Bama miniature pigs (6 months of age; 12–15 kg) were used in this study. The animals were randomly assigned to two groups: a control group (Con, *n* = 4) fed a standard chow diet, and an atherosclerosis model group (AS, *n* = 8) fed a HCFD (HCFD was procured from Beijing Ke'ao Xieli Feed Co., Ltd., including 2% cholesterol, 15% beef tallow, 0.5% bile salt, and 82.5% chow diet) for 9 consecutive months to induce atherosclerotic lesions.

### 2.2 Measurement of body weight and serum lipid profiles

Body weight and serum lipid levels were monitored monthly throughout the 9‐month study. Serum lipid analysis, which included triglyceride (TG), LDL‐C, high‐density lipoprotein cholesterol (HDL‐C), total cholesterol (TC), was performed by Beijing North Institute of Biotechnology Co., Ltd.

### 2.3 Proteomic analysis

#### 2.3.1 Sample preparation and digestion

Coronary artery tissue samples were homogenized in lysis buffer and subsequently sonicated. The lysate was clarified through centrifugation at 13 000 rpm for 20 min at 4°C. Proteins were precipitated from the supernatant overnight at −20°C using a sixfold volume of acetone. The resulting protein pellet was washed, air‐dried, and redissolved in a buffer containing 300 mmol/L triethylamine borane (TEAB) and 6 mol/L guanidine hydrochloride. Protein concentration was quantified using a bicinchoninic acid (BCA) assay.

Protein digestion was performed following the filter‐aided sample preparation (FASP) protocol. Briefly, 100 μg of protein from each sample was reduced with 20 mmol/L dithiothreitol (DTT) for 1 h at 37°C, followed by alkylation with 90 mmol/L iodoacetamide for 40 min at room temperature in the dark. The samples were then loaded onto 10 kDa molecular weight cutoff tubes, washed four times with TEAB, and digested overnight at 37°C with trypsin. The resulting peptides were collected using centrifugation and dried via centrifugal concentration.

#### 2.3.2 TMT labeling and high‐pH reversed‐phase fractionation

Dried peptides were redissolved in TEAB, and labeling was performed using two TMT 10‐plex Isobaric Label Reagent Sets (Thermo Scientific, USA) via a bridging approach according to the manufacturer's instructions. A pooled sample, created by combining aliquots from all samples, was included as an internal reference for batch adjustment. After being labeled, all samples were combined, desalted using a MonoSpin C18 column, and dried. The TMT‐labeled peptide mixture was then fractionated using a high‐pH reversed‐phase UPLC system (Waters), and the collected fractions were concatenated into 10 final fractions for subsequent analysis.

#### 2.3.3 Liquid chromatography tandem mass spectrometry

The dried sample was dissolved in buffer (0.1% formic acid [FA]); each fraction was analyzed using a Q Exactive HF‐X Hybrid Quadrupole‐Orbitrap mass spectrometer coupled to an EASY‐nLC 1200 system (Thermo Scientific). The analytical column used was a C18 reversed‐phase column (C18, 2 μm, 75 μm × 250 mm). Peptides were eluted from the trap column at a flow rate of 300 nL/min using an increasing concentration gradient of buffer (0.1% formic acid in acetonitrile) over a duration of 120 min. The peptide samples were analyzed using a nano electrospray ionization source coupled with tandem mass spectrometry on the Orbitrap Exploris 480 (Thermo Scientific). The mass spectrometer operated in data‐dependent mode, with MS2 scans covering a mass‐to‐charge ratio (*m/z*) range of 350 to 1600. Intact peptides were detected at a resolution of 120 000, with a collision energy of 35% for higher‐energy collisional dissociation (HCD).

#### 2.3.4 Proteomic data analysis

Raw mass spectrometry data were processed using Proteome Discoverer (version 2.4, Thermo Scientific). The spectra were analyzed against the UniProt Sus scrofa (pig) protein database (June 2020 release) employing the SEQUEST HT search engine. The search parameters included trypsin as the enzyme, allowing for a maximum of two missed cleavages, with carbamidomethylation of cysteine designated considered as a fixed modification, and oxidation of methionine along with *N*‐terminal acetylation as variable modifications. Differentially expressed proteins (DEPs) were identified based on a fold change threshold of ≥1.2 (or ≤0.833) and a *p*‐value of <0.05.^20^ Gene ontology (GO) and KEGG pathway enrichment analyses were performed using the “ClusterProfiler” R package.

#### 2.3.5 Validation by multiple reaction monitoring

A targeted proteomic approach using multiple reaction monitoring (MRM) was employed for validation. Based on the DDA spectral library, optimal peptide transitions were selected using Skyline software (version 21.1). At least three transitions per peptide were monitored using a QTRAP 6500+ mass spectrometer (AB Sciex). Data were imported into Skyline for visualization and quantification, where peptide abundance was calculated as the mean summed peak area of its selected transitions. Indexed retention time (iRT) standards (Biognosys AG) were added to all samples for retention time calibration and signal stability monitoring.^21^

### 2.4 PPI network construction and hub protein identification

A PPI network of the DEPs was constructed using the STRING database (version 11.5, https://cn.string‐db.org/) with a minimum required interaction score of 0.4. The network visualization was performed using Cytoscape software (version 3.9.1). To identify central nodes, five distinct topological algorithms within the CytoHubba plugin were employed: maximal clique centrality (MCC), maximum neighborhood component (MNC), Degree, maximum neighborhood component centrality (DMNC), and EcCentricity.^22^ Proteins consistently ranking in the top 10 across all selected algorithms were designated as high‐confidence hub proteins, and their overlap was visualized using a Venn diagram.

### 2.5 Histology and immunostaining

For morphological assessment, sections were stained with hematoxylin and eosin (H&E) according to standard protocols.^23^ For immunohistochemistry (IHC) and immunofluorescence (IF), sections were incubated with the following primary antibodies: anti‐CLU (Affinity Biosciences, cat#: DF6421), anti‐CD31 (for ECs; Proteintech, cat#: 66065‐2‐Ig), anti‐α‐SMA (for SMCs; Abcam, cat#: ab7817).

For immunofluorescence, an Alexa Fluor 594‐conjugated secondary antibody (Bioss, cat#: Bs‐0295D‐AF594) was used, and sections were mounted with an anti‐fade medium containing DAPI (YangGuangBio, cat#: C190401) for nuclear counterstaining. All staining procedures were performed according to manufacturers' instructions.^7^

### 2.6 Western blot

Total protein was extracted from porcine coronary tissues using RIPA buffer (Aoqing Biotechnology), which was supplemented with protease and phosphatase inhibitors.^24^ Protein concentrations were determined using a BCA protein assay kit (YangGuangBio). Equal amounts of protein per lane were separated using SDS‐PAGE and subsequently transferred onto nitrocellulose membranes. The membranes were blocked and incubated overnight at 4°C with the following primary antibodies: anti‐CLU (Affinity, DF6421), anti‐LRP1 (Affinity, DF2935), anti‐phospho‐AKT (S473) (Abcam, ab81283), anti‐total AKT (Abcam, ab32505), and anti‐β‐actin (loading control; Affinity, AF7018). After this, the membranes were incubated with appropriate HRP‐conjugated secondary antibodies for 1 h at room temperature. Protein bands were visualized using a ChemiDoc XRS+ Imaging System (Bio‐Rad), and band intensities were quantified using ImageJ software.

### 2.7 Key proteins diagnosis model construction

To evaluate the diagnostic potential of each key protein, we performed receiver operating characteristic (ROC) curve analysis on the original datasets using the pROC package in R. The diagnostic accuracy was quantified by calculating the area under the curve (AUC), with an AUC greater than 0.7 considered indicative of acceptable diagnostic performance.

### 2.8 AS mouse model

Reasonable acquisition of AS mouse (*Apoe* ^*−/−*^ mice: a control group [Con, *n* = 3] fed a standard chow diet, and an atherosclerosis model group (AS, *n* = 3) fed a HCFD for 10 consecutive months to induce atherosclerotic lesions) was obtained from Professor Li Zhang of the Institute of Laboratory Animal Science, Chinese Academy of Medical Sciences & Peking Union Medical College.

### 2.9 Statistical analysis

All quantitative data are expressed as the mean ± standard deviation (SD) and are derived from a minimum of three independent biological replicates. For imaging analysis, parameters, including the number of double‐positive cells and positive area fraction, were quantified using Fiji (an ImageJ distribution). Statistical comparisons between two groups were performed using an unpaired, two‐tailed Student's *t*‐test in GraphPad Prism (version 9.0). A *p*‐value <0.05 was deemed statistically significant.

## 3 RESULTS

### 3.1 HCFD induces an atherosclerotic phenotype in Bama miniature pigs

**FIGURE 1:**
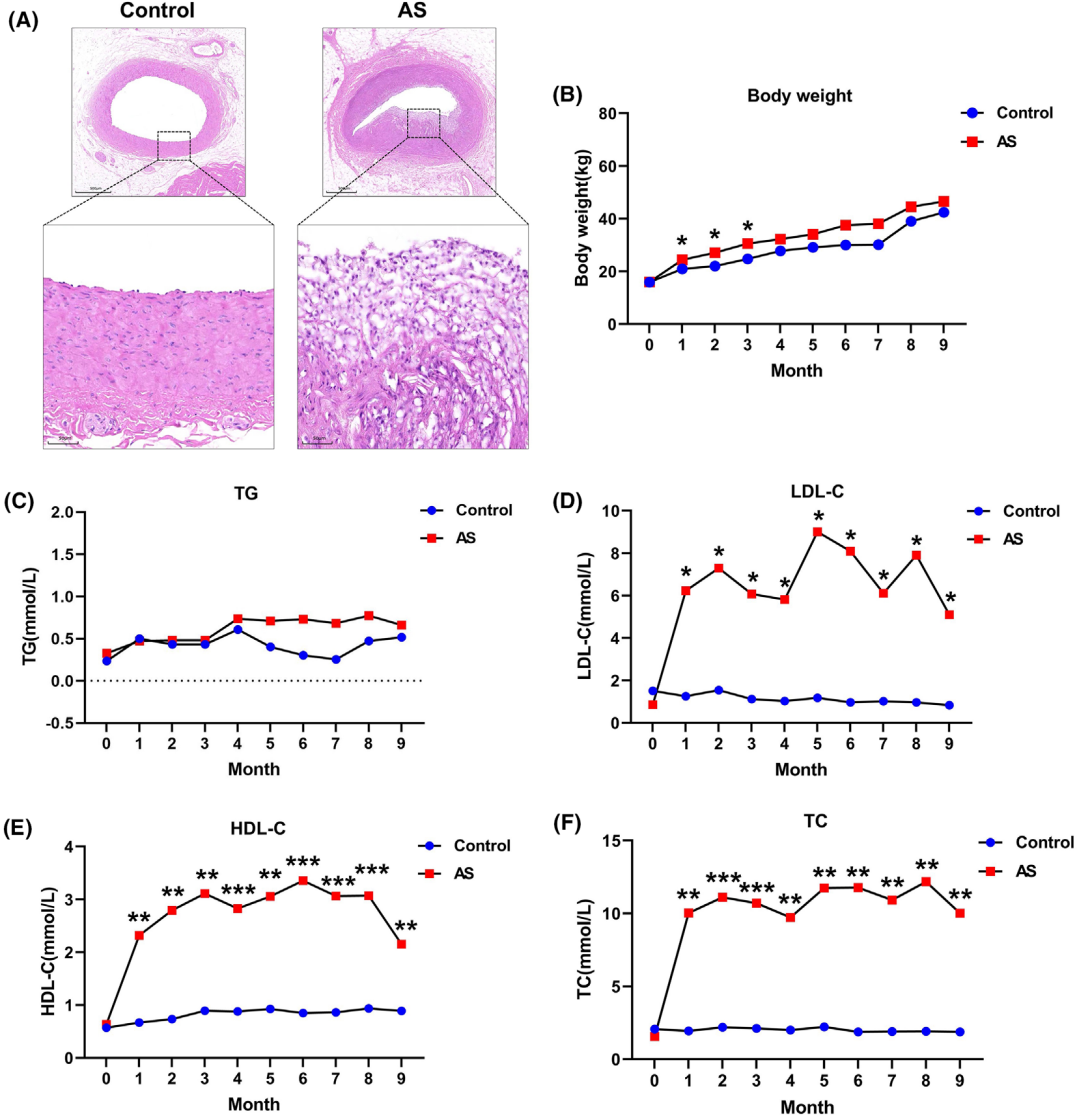
High‐cholesterol, high‐fat diet (HCFD) induces an atherogenic phenotype in the porcine model. (A) Representative hematoxylin and eosin (H&E)‐stained sections of coronary arteries from control and AS pigs. Monthly progression of (B) body weight, (C) serum triglycerides (TG), (D) low‐density lipoprotein cholesterol (LDL‐C), (E) high‐density lipoprotein cholesterol (HDL‐C), and (F) total cholesterol (TC) in control (Con, *n* = 4) and atherosclerosis (AS, *n* = 8) pigs. Data are presented as mean ± standard deviation (SD). *p < 0.05, **p < 0.01, ***p < 0.001 versus control group, determined by an unpaired, two‐tailed Student's *t*‐test.

After the 9‐month dietary intervention, H&E staining confirmed the presence of advanced atherosclerotic lesions in the coronary arteries of pigs subjected to atherogenic diets. Compared to the control group, pigs in the HCFD group exhibited significantly higher body weights, with the most pronounced differences observed during the first 3 months. Consistent with the development of an atherosclerotic phenotype, serum levels of TC, HDL‐C, and LDL‐C were significantly elevated in the HCFD group relative to the control group. In contrast, serum TG levels showed no significant difference between the two groups. No mortality was observed in either group throughout the study period. These results are summarized in Figure 1.

### 3.2 Proteomic analysis reveals key pathways involved in AS

**FIGURE 2:**
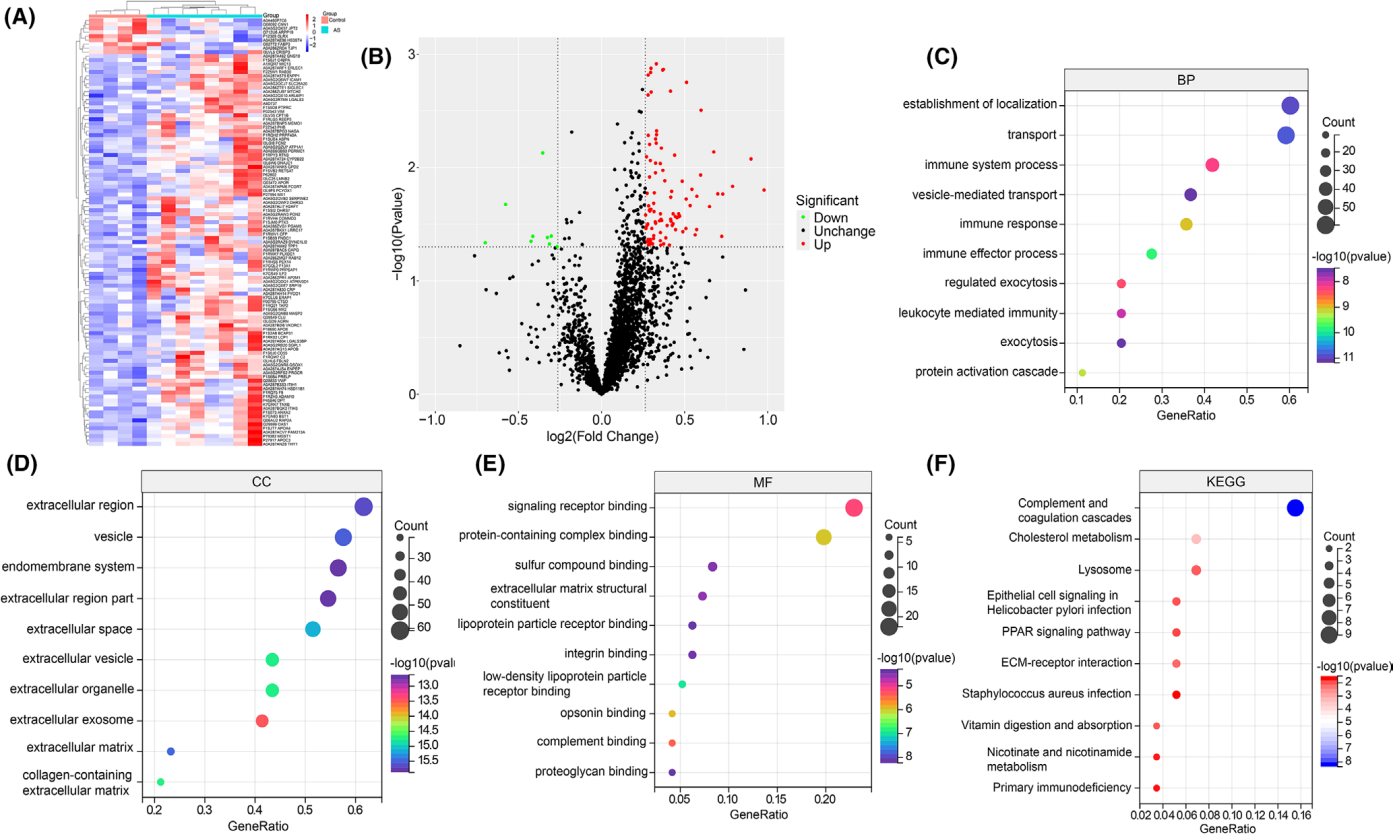
Proteomic analysis of coronary arteries identifies key pathways in atherosclerosis. (A) Hierarchical clustering heatmap of 108 differentially expressed proteins (DEPs) between control (Con) and AS groups. (B) Volcano plot illustrating DEPs. Red dots represent upregulated proteins; green dots represent downregulated proteins. Significance threshold (dashed lines) was set at |fold change| ≥1.2 and a *p*‐value <0.05. (C–F) Gene ontology (GO) and KEGG pathway enrichment analyses of the DEPs, showing the most significant terms for (C) biological process, (D) cellular component, (E) molecular function, and (F) KEGG pathways.

To identify the molecular changes associated with AS development, we performed a quantitative proteomic analysis of coronary artery tissues from both control (*n* = 4) and HCFD (*n* = 8) pigs. A total of 2609 proteins were identified across all samples. Applying a cutoff of |fold change| ≥1.2 and a *p*‐value <0.05, we identified 108 DEPs. Among these, 99 proteins were upregulated and 9 were downregulated in the HCFD group, as illustrated in the heatmap and volcano plot (Figure 2A,B). A full list of DEPs is provided in Table S1.

To elucidate the biological functions of these DEPs, we performed GO and KEGG pathway enrichment analyses. The GO analysis showed significant enrichment in terms related to biological processes (BP, establishment of localization, transport, and the immune system processes), cellular components (CC, the extracellular region, vesicles, and the endomembrane system), molecular functions (MF, signaling receptor binding, protein‐complex binding, and sulfur compound binding). Furthermore, KEGG analysis highlighted pathways highly relevant to AS, most notably the complement and coagulation cascades, cholesterol metabolism, and lysosome pathways (Figure 2C–F).

### 3.3 PPI network analysis and identification of hub proteins in AS

**FIGURE 3:**
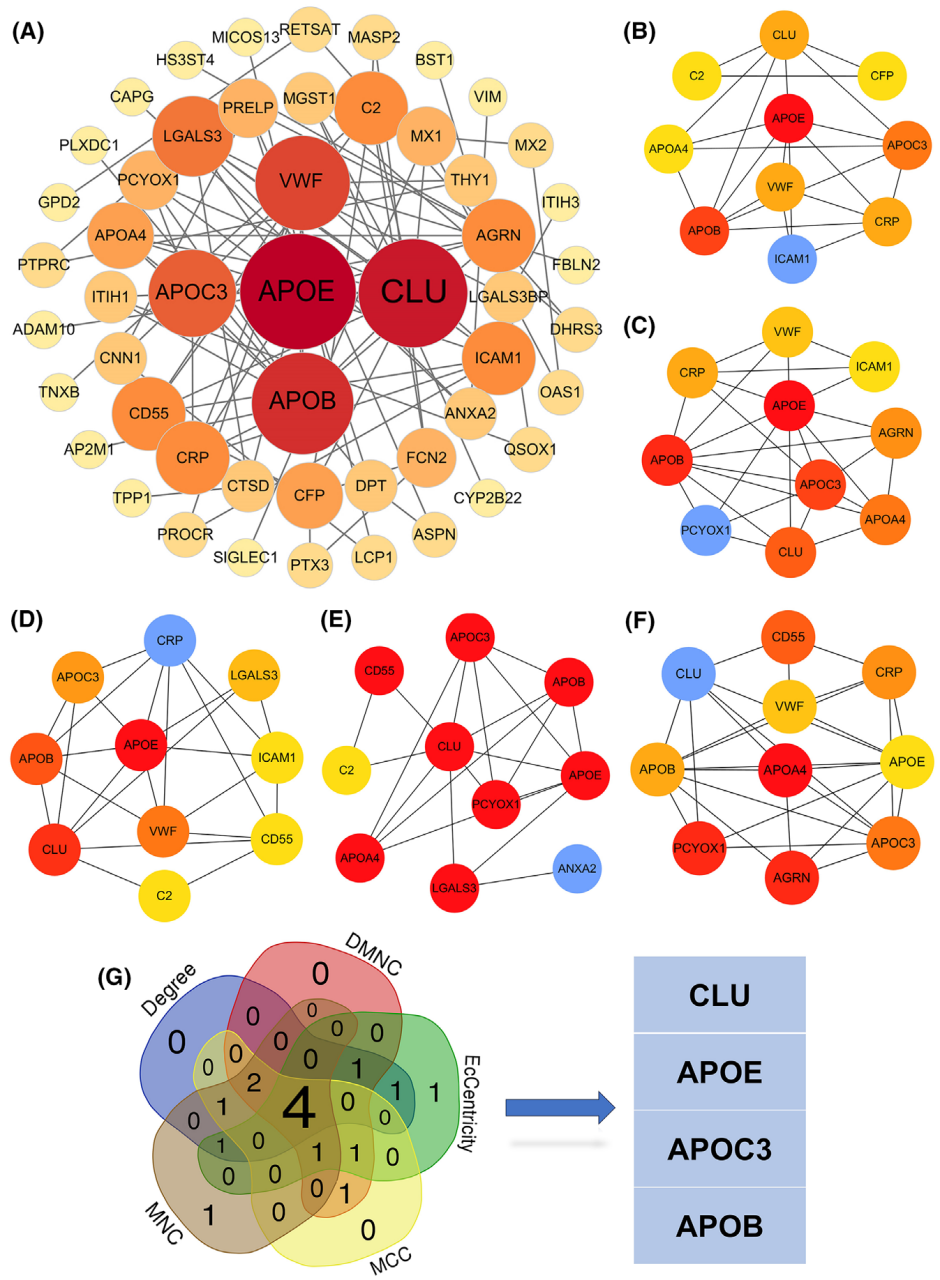
Bioinformatic analysis of the protein–protein interaction (PPI) network reveals four hub proteins. (A) PPI network of the differentially expressed proteins (DEPs) constructed using the STRING database and Cytoscape. (B–F) The top 10 ranked proteins from five different CytoHubba algorithms: (B) maximal clique centrality (MCC), (C) maximum neighborhood component centrality (DMNC), (D) maximum neighborhood component (MNC), (E) Degree, and (F) EcCentricity. (G) A Venn diagram showing the intersection of the top‐ranked proteins, which identified four common hub proteins.

To map the functional relationships among the 108 DEPs, we constructed a PPI network using the STRING database. The results of the network analysis results were subsequently imported using Cytoscape software for further refinement. This selection process revealed a total of 51 core targets, and the corresponding PPI network was visualized (Figure 3A). To identify the most critical nodes within the PPI network, the CytoHubba plugin in Cytoscape was employed to rank all proteins using five distinct topological algorithms (MCC, MNC, Degree, DMNC, and EcCentricity). By intersecting the top 10 proteins from each algorithm, we identified a core set of four high‐confidence hub proteins: apolipoprotein B (APOB), apolipoprotein E (APOE), apolipoprotein C3 (APOC3), and CLU (Figure 3B–G).

### 3.4 Hub protein expression is upregulated in atherosclerotic tissues and diagnosis model construction

**FIGURE 4:**
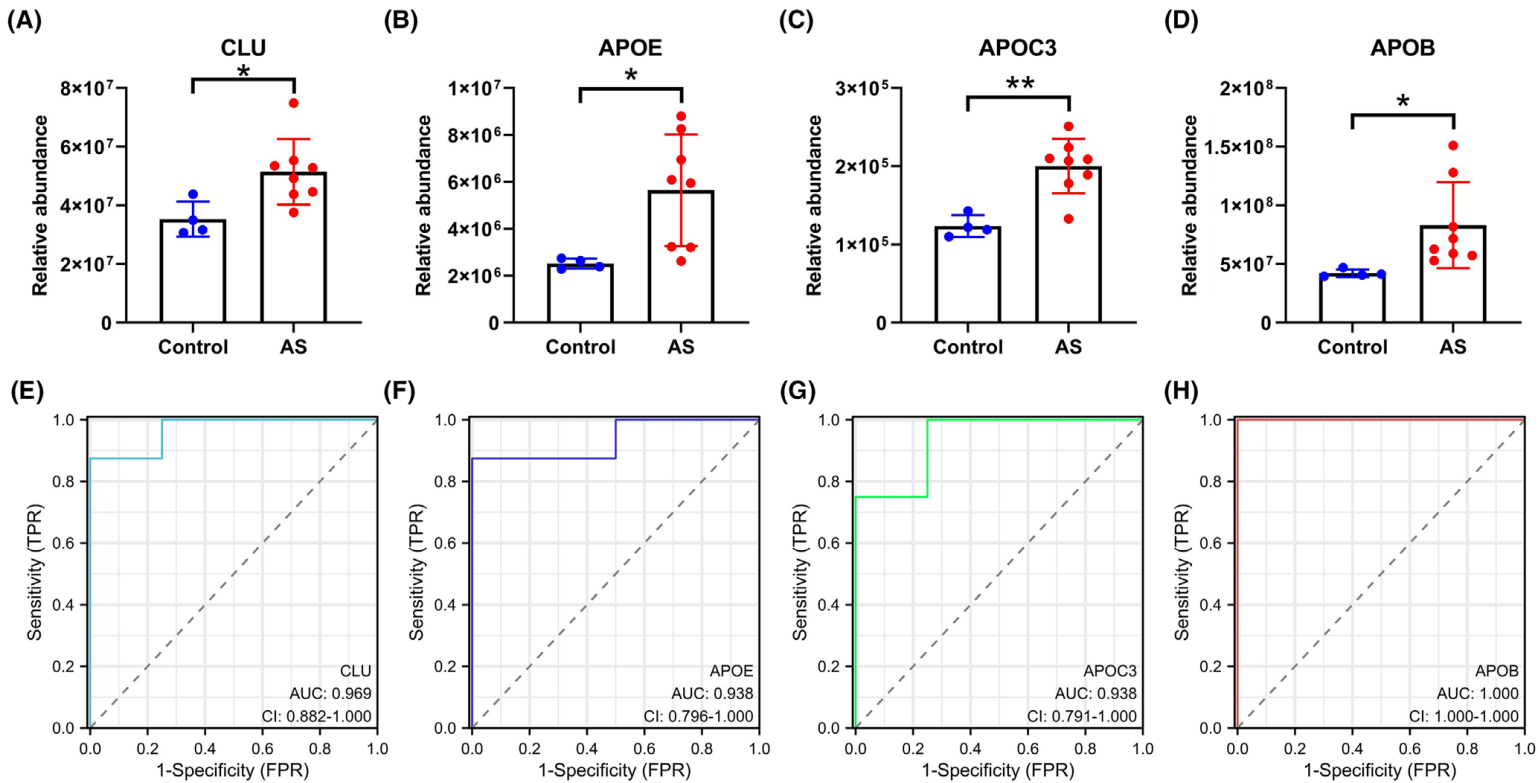
Validation of hub protein abundance in coronary tissues and diagnosis model construction. Relative protein abundance of (A) APOB, (B) APOC3, (C) APOE, and (D) Clusterin (CLU) in the coronary arteries of control (Con) and AS pigs, as determined by proteomic analysis. (E–H) Receiver operating characteristic (ROC) curves evaluating the diagnostic accuracy of the four hub proteins in the original dataset. Data are presented as mean ± standard deviation (SD). *p < 0.05, **p < 0.01 versus Con.

To validate these bioinformatic findings, we analyzed the quantitative proteomic data for the four identified hub proteins. The results indicated that the expression levels of CLU, APOC3, APOE, and APOB were significantly higher in the coronary artery tissues of the HCFD group compared to those of the control group (Figure 4A–D). Next, we assessed the diagnostic potential of these proteins using ROC curve analysis on the original datasets. In the single‐protein diagnostic model, this analysis revealed that four hub proteins—CLU, APOE, APOC3, and APOB—had a significant diagnostic value for AS. Notably, all the four hub proteins exhibited good performance, with an AUC of 0.969 (CLU), 0.938 (APOE), 0.938 (APOC3), and 1.000 (APOB) (Figure 4E–H). These findings suggest that these four proteins could serve as effective biomarkers for detecting AS. This finding demonstrates that these key proteins are robustly upregulated during the progression of diet‐induced AS.

### 3.5 CLU is upregulated in ECs and SMCs of atherosclerotic lesions

Although the roles of APOB, APOE, and APOC3 in AS are well documented, the function of CLU remains less defined. Therefore, we focused our subsequent investigations on elucidating the role of CLU in AS. We validated its expression in coronary artery tissues from both the porcine HCFD model and an independent murine model of AS.

**FIGURE 5:**
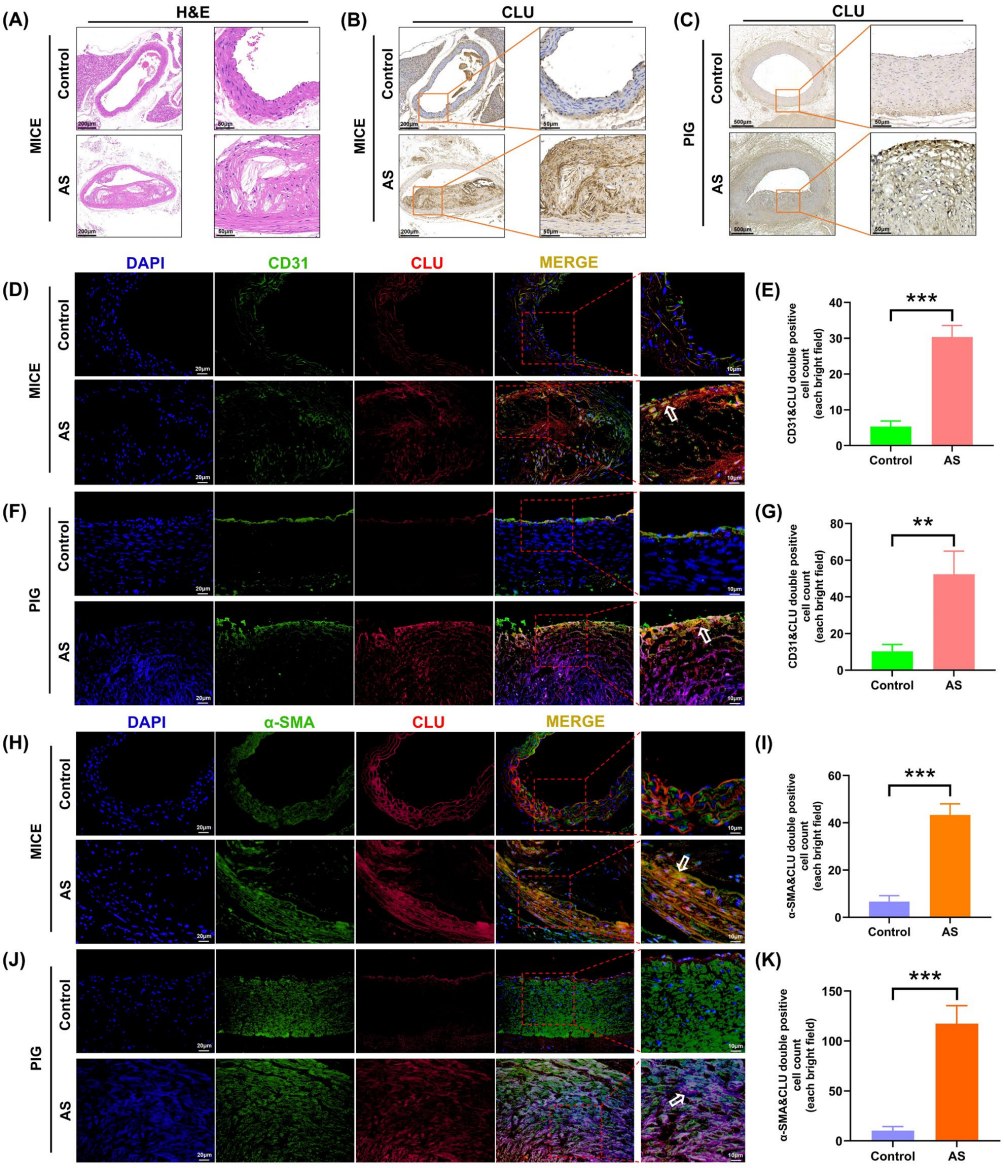
Clusterin (CLU) is upregulated and localized to endothelial and smooth muscle cells in atherosclerotic arteries. (A) Representative hematoxylin and eosin (H&E)‐stained sections of coronary arteries from control and AS mice. (B, C) Immunohistochemistry showing increased CLU staining in atherosclerotic plaques. (D–G) Dual immunofluorescence for CLU (red) and the endothelial cell marker CD31 (green). White arrows indicate colocalization. (H–K) Dual immunofluorescence for CLU (red) and the smooth muscle cell marker α‐SMA (green). Bar graphs show quantification of double‐positive cells. Nuclei were counterstained using DAPI (blue). Data are presented as mean ± standard deviation (SD). **p < 0.01, ***p < 0.001. Scale bars are provided in the images.

As expected, H&E staining confirmed the presence of advanced atherosclerotic lesions in the coronary arteries of mice subjected to atherogenic diets, compared to Figure 5A. Immunohistochemical analysis revealed that CLU expression was significantly increased within these atherosclerotic plaques (Figure 5B,C).

To identify the specific cell types expressing CLU, we performed dual‐label immunofluorescence. In the intima of atherosclerotic arteries, a significant increase was observed in cells coexpressing CLU and the EC marker CD31. Similarly, in the medial layers, there was a significant increase in cells coexpressing CLU and the SMC marker α‐SMA (Figure 5D–K). These findings demonstrate that CLU is robustly upregulated in both ECs and SMCs within atherosclerotic lesions.

### 3.6 CLU upregulation is associated with LRP1/AKT pathway activation

To explore the molecular mechanism downstream of CLU, we performed Western blot analysis on protein lysates from porcine coronary arteries. The analysis first confirmed the significant upregulation of CLU protein in the HCFD group.

**FIGURE 6:**
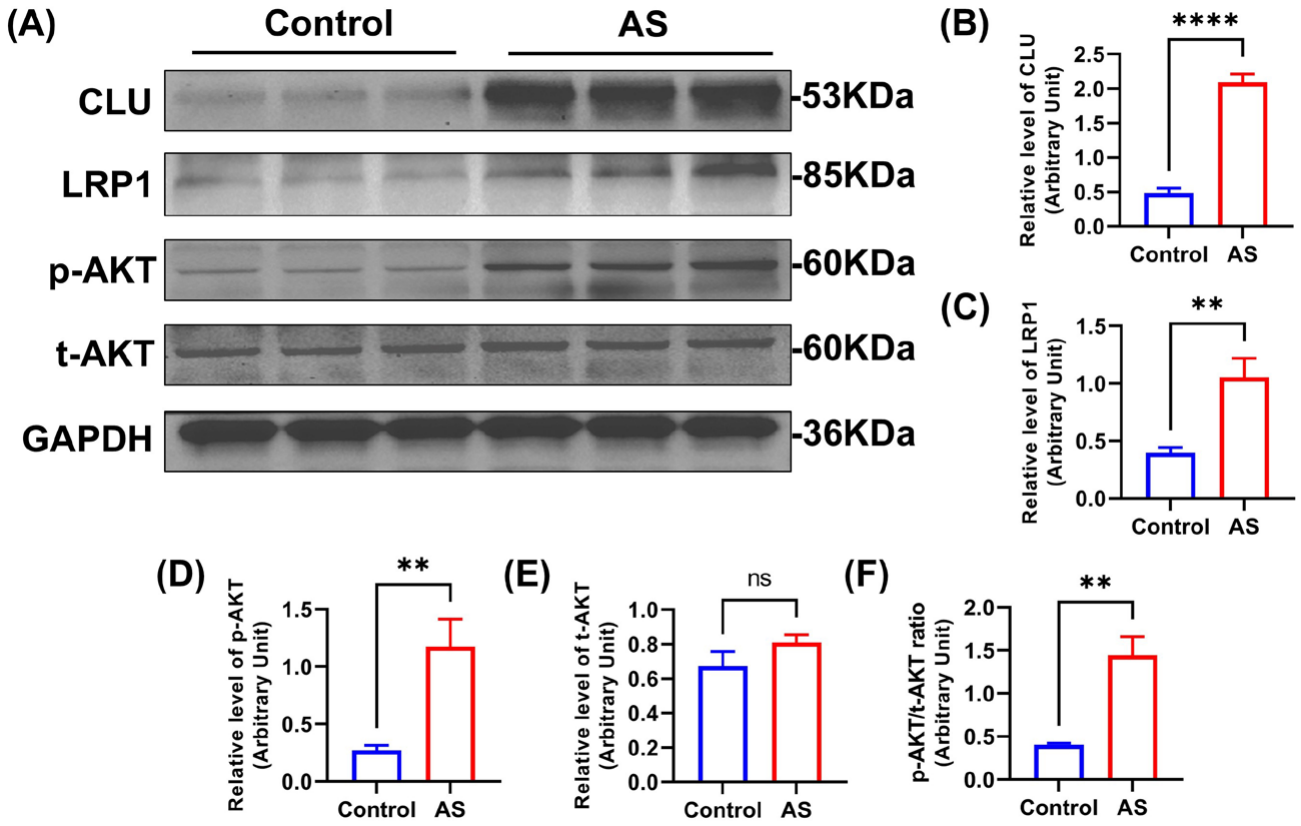
Clusterin (CLU) upregulation is associated with LRP1/AKT pathway activation. (A) Representative Western blots of CLU, LRP1, phosphorylated AKT (p‐AKT, S473), and total AKT in coronary artery lysates. β‐actin served as the loading control. (B–F) Densitometric quantification of the Western blot bands for (B) CLU, (C) LRP1, (D) the p‐AKT/total AKT ratio, (E) total AKT, and (F) p‐AKT. Data are presented as mean ± standard deviation (SD). ns, not significant, **p < 0.01, ****p < 0.0001 versus control group.

Crucially, this increase in CLU was accompanied by a significant elevation in the protein levels of LDL receptor‐related protein 1 (LRP1), which is highly consistent with the conclusion reported by Yang Tian et al.^25^ that CLU significantly elevates LRP1 protein levels through synergistic interaction with extracellular heat shock protein 90α (eHsp90α). Furthermore, we observed a marked increase in the phosphorylation of AKT at serine 473, a key indicator of its activation. Consequently, the ratio of phosphorylated AKT to total AKT was significantly higher in the HCFD group (Figure 6A–F). Increased expression of LRP1 has been reported to activate multiple signaling pathways, including AKT, ERK, and NF‐κB.^25^, ^26^ In this study, we observed the activation of LRP1/AKT in the HCFD group. Overall, these data reveal a strong association between CLU upregulation and the activation of the LRP1/AKT signaling pathway in atherosclerotic tissue. The complete Western blot images are provided in Supplementary Data.

## 4 DISCUSSION

In this study, we utilized a porcine model of diet‐induced AS, which closely recapitulates human pathology, to investigate the molecular drivers of the disease. Using quantitative proteomics of coronary artery tissues, we identified CLU as a key upregulated protein in advanced atherosclerotic lesions and proceeded to elucidate its pro‐atherogenic role and underlying mechanisms.

The choice of an animal model is paramount for the translational relevance of AS research. Although rodent models are widely used, their lipid metabolism differs significantly from humans. In contrast, miniature pigs offer profound similarities in lipoprotein profiles and the anatomical distribution and complexity of atherosclerotic plaques, making them an invaluable tool.^27^, ^28^ Our observation of significantly elevated TC and LDL‐C in HCFD‐fed pigs aligns with classic atherogenic dyslipidemia.^29^, ^30^ However, an increase in HDL‐C concentration was observed, which is consistent with the findings from previous observations in pigs^31^, ^32^, ^33^ and other diet‐induced hyperlipidemia^34^ animal models. We speculate that it may be due to a significant increase in TC. Studies have shown that feeding pigs with HCFD can lead to the emergence of β–VLDL‐C (a cholesterol‐rich lipoprotein containing APOE) and HDL‐C.^35^ Although pigs plasma lack cholesterol ester transfer protein (CETP), they can unidirectionally transfer cholesterol esters from high‐density lipoprotein to low‐density lipoprotein.^36^ Interestingly, we also observed that although the body weight of pigs in the AS group was higher than that in the Con group between 4 and 9 months, the difference was not statistically significant, which is because miniature pigs, as large experimental animals, still exhibit natural genetic heterogeneity in individual metabolic efficiency and energy absorption capacity.^37^ With sample sizes of three and six pigs per group in the experiment, the limited sample size may have diluted the statistical power of intergroup differences to some extent due to individual weight variations.

However, a notable limitation of the present study lies in the exclusive use of 12 male Bama miniature pigs as experimental subjects, which prevents us from addressing the potential impact of gender differences on AS development in this animal model. Indeed, accumulating evidence has indicated that gender‐related biological factors, including sex hormone levels, immune response profiles, and lipid metabolism patterns, play critical roles in modulating the initiation and progression of AS.^38^, ^39^, ^40^ For instance, estrogen has been reported to exert atheroprotective effects by inhibiting vascular smooth muscle cell proliferation and reducing pro‐inflammatory cytokine secretion,^41^, ^42^ whereas testosterone may promote lipid accumulation in atherosclerotic lesions under certain conditions.^43^ In the human population, female patients typically exhibit less‐severe atherosclerotic plaques than their male counterparts within the same age group, which highlights the necessity of investigating gender dimorphism in AS research.^43^ Given this gap, we are currently conducting a follow‐up study with 40 pigs (20 males, 20 females) to specifically address this variable. The existing results of this follow‐up study are expected to provide direct experimental evidence for the gender dimorphism in AS development in Bama miniature pigs.

In this study, we obtained 108 DEPs using quantitative proteomic analysis, and then analyzed these DEPs using CytoHubba plugins to identify key factors. Finally, CLU is considered to be a key protein associated with the progression of coronary AS in miniature pigs. Our unbiased proteomic screen pinpointed CLU as a hub protein of high interest. CLU is a notoriously pleiotropic chaperone whose function is highly context‐dependent.^12^, ^16^, ^44^, ^45^ The expression of this factor is regulated by genetic factors, various organs, additional genes, xenobiotics, signal transduction pathways, and pathological states.^12^, ^46^, ^47^, ^48^ This suggests that it may function as a sensor for alterations in cellular function and dynamic balance. Our study revealed that CLU is upregulated in coronary artery samples from different AS disease models, including porcine coronary arteries (HCFD food induced for 9 months) and mouse coronary arteries (*Apoe* ^*−/−*^ mice induced with high‐fat and high‐cholesterol diet for 10 months). These findings support the hypothesis that CLU was involved in disease progression by changing cell function and cell dynamic balance in AS.^49^, ^50^ Although previous reports have detected CLU in early human atherosclerotic lesions^51^ and suggested a protective role,^18^, ^52^ its precise function during the progression of advanced AS has remained ambiguous. Our study addresses this gap by demonstrating robust CLU upregulation in advanced coronary lesions across two distinct mammalian models—diet‐induced porcine AS and genetic (*Apoe* ^*−/−*^) murine AS.

A key finding of our study is the specific localization of this upregulated CLU within both ECs and SMCs of the atherosclerotic vessel wall. This cellular distribution provides critical insight into its potential pro‐atherogenic function. Previous in vitro studies have shown that CLU can stimulate SMC proliferation and migration—key events in forming the neointima.^50^, ^53^ Our results provide strong in vivo evidence supporting this role, as CLU‐positive SMCs were prominent in the thickened intima of atherosclerotic plaques. Similarly, the upregulation of CLU in ECs suggests a role in endothelial dysfunction, a notion supported by reports that silencing CLU inhibits endothelial migration and angiogenesis.^54^

**FIGURE 7:**
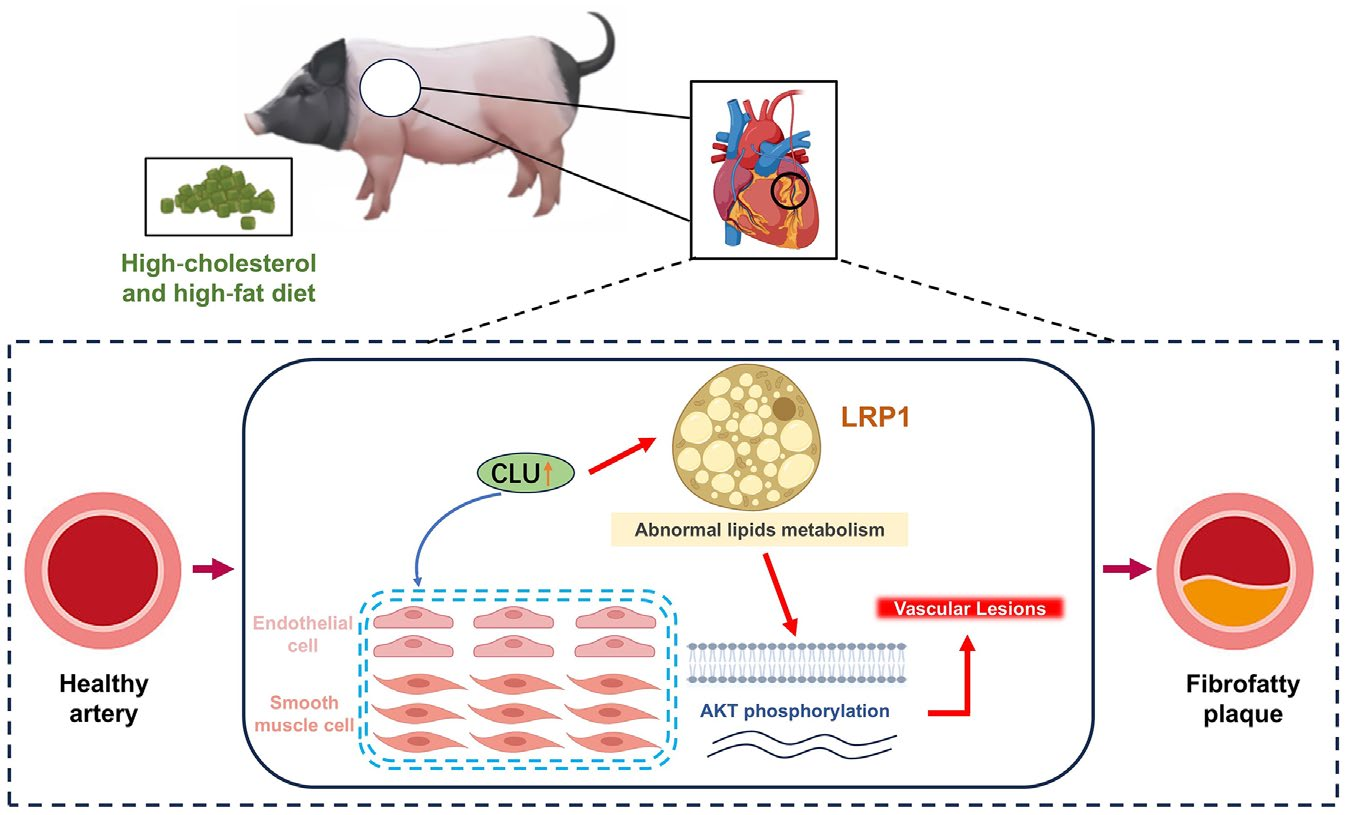
A proposed model for the pro‐atherogenic role of clusterin (CLU). This schematic illustrates the proposed mechanism. In advanced atherosclerotic lesions, upregulated CLU on the cell surface activates low‐density lipoprotein (LDL) receptor‐related protein 1 (LRP1). This interaction triggers the phosphorylation and activation of AKT. The activated CLU/LRP1/AKT signaling axis is proposed to promote pathological processes in endothelial and smooth muscle cells, such as inflammation and mesenchymal‐like transformation, thereby collectively accelerating atherosclerotic plaque progression.

To uncover the mechanism downstream of CLU, we investigated key signaling pathways. Our results revealed that the upregulation of CLU in atherosclerotic tissues strongly correlated with increased expression of LRP1 and subsequent phosphorylation‐mediated activation of AKT. This CLU/LRP1/AKT axis provides a plausible molecular basis for the observed pathology. It has been reported that CLU synergizes with extracellular heat shock protein 90α (eHsp90α) to promote epithelial‐mesenchymal transition (EMT) and metastasis in breast cancer, and this regulatory effect is dependent on LRP1. Specifically, in in vitro experiments using the human breast cancer MCF‐7 cell line, overexpression of both CLU and eHsp90α proteins was found to induce a significant upregulation of LRP1 protein expression. Mechanistically, CLU enhances the binding affinity of eHsp90α to LRP1 by participating in the formation of the eHsp90α‐LRP1 complex, thereby activating downstream signaling pathways such as AKT, ERK, and NF‐κB to drive EMT and metastatic progression of breast cancer cells,^25^, ^55^ a process sharing molecular hallmarks with the pathological phenotype switching of SMCs in AS.^56^ Therefore, we speculate that CLU may mediate the mesenchymal transformation of SMCs through LRP1 in AS. Accumulating evidence indicates that LRP1‐driven AKT activation constitutes a conserved mechanism contributing to the malignant phenotype of several cancer types.^57^ For instance, in osteosarcoma, recurrent LRP1 fusion genes have been shown to promote tumor cell motility by activating the AKT pathway and upregulating matrix metalloproteinases (MMPs), which facilitate extracellular matrix degradation and tumor invasion.^58^ Notably, LRP1 itself has also been implicated in restraining cellular inflammatory responses; its dual role in maintaining cholesterol homeostasis and modulating inflammation was recently validated using an adipocyte‐specific LRP1‐deficient mouse model^59^; at the early stage of the AS, LRP1 exerts atheroprotective effects by modulating hepatocyte‐mediated clearance of cholesterol‐rich remnant lipoproteins from the circulation.^60^ Regrettably, despite the initial effect of LRP1 phosphorylation in reducing macrophage cholesterol accumulation, activation of downstream pathways, including PI3K/AKT by LRP1 during the intermediate and advanced stages of AS, contributes to the exacerbation of atherosclerotic plaque progression.^61^ Meanwhile, we found that increased CLU protein levels in AS group could activate LRP1, further causing AKT phosphorylation. Collectively, these findings provide robust support for the functional role of the CLU/LRP1/AKT signaling axis in the pathogenesis of AS in our study. Therefore, we propose a model where elevated CLU in the arterial wall activates LRP1, triggering AKT‐dependent signaling that promotes detrimental SMC proliferation and migration while exacerbating endothelial inflammation, thereby collectively accelerating atherosclerotic progression (Figure 7).

Although this study provides compelling evidence for the pro‐atherogenic role of the CLU/LRP1/AKT axis, we acknowledge its limitations. Our findings are based on associative evidence from in vivo models. Future research employing in vitro gain and loss‐of‐function studies in primary ECs and SMCs is necessary to definitively establish a causal relationship and dissect the precise molecular events governed by CLU in each cell type. Additionally, we are currently conducting a follow‐up study that include both male and female Bama miniature pigs to systematically evaluate gender‐specific differences in the susceptibility to coronary AS, as well as the expression and regulatory function of CLU in lesions of different genders. Such comparative analyses will not only enhance the translational relevance of our findings but also provide empirical evidence for developing gender‐tailored preventive and therapeutic strategies against AS.

## 5 CONCLUSIONS

Our study identifies CLU as a key pro‐atherogenic factor that accelerates the development of AS. We provide compelling evidence that elevated CLU expression, particularly within vascular ECs and SMCs, exacerbates the disease by activating the LRP1/AKT signaling pathway. This mechanism likely contributes to plaque formation by promoting pathological events such as the mesenchymal transformation of SMCs and endothelial inflammation. These findings not only advance our fundamental understanding of diet‐induced AS but also establish the CLU/LRP1/AKT axis as a promising therapeutic target for this critical cardiovascular disease.

## AUTHOR CONTRIBUTIONS

**Dengfeng Ding:** Conceptualization; methodology; software; validation; visualization; writing – original draft. **Yingjie Zhang:** Formal analysis; methodology; software; validation. **Li Zhang:** Validation; writing – review and editing. **Xinou Zheng:** Methodology; software. **Yunxiao Jia:** Data curation. **Chao Guo:** Funding acquisition; supervision. **Tao Jiang:** Conceptualization; validation; visualization; writing – original draft; writing – review and editing. **Yuqiong Zhao:** Conceptualization; funding acquisition; methodology; project administration; software; supervision; validation; visualization; writing – original draft; writing – review and editing. **Miaomiao Niu:** Supervision. **Xuezhuang Li:** Resources; validation. **Hua Chen:** Conceptualization; funding acquisition; resources; supervision.

## FUNDING INFORMATION

This work was supported by the National Natural Science Foundation of China, no. 32370568.

## CONFLICT OF INTEREST STATEMENT

The authors declare that they have no competing interests. All authors have reviewed and approved the final version of the manuscript.

## ETHICS STATEMENT

All animal experiments were conducted in accordance with the guidelines established by the Institutional Animal Care and Use Committee of the Chinese PLA General Hospital (ID: 2018‐D14‐26).

## Supporting information


Data S1.

**Table S1.** List of 108 differential expression proteins.

## Data Availability

The original contributions presented in this study are included in the article and the Supplementary Material. And further inquiries can be directed to the corresponding author.
