# Supplementary material for: Quantitative proteomics identifies clusterin as a novel biomarker for atherosclerosis
Source: Animal Model Exp Med. 2026 Feb 2;9(4):685–97. doi: 10.1002/ame2.70143 (PMC13242730; doi:10.1002/ame2.70143)
Supplement: Supplementary file 1 — Data S1. [file AME2-9-685-s002.docx]

**Quantitative Proteomics Identifies Clusterin as a Novel Biomarker and Therapeutic Target for Atherosclerosis**

Dengfeng Ding^1#^, Yingjie Zhang^2#^, Li Zhang^3^, Xinou Zheng^1^, Miaomiao Niu^1^, Yunxiao Jia^1^, Xuezhuang Li^1^, Hua Chen^1^, Chao Guo^1^, Tao Jiang^1*^, Yuqiong Zhao^1*^

^1^Medical Innovation Research Department, Chinese PLA General Hospital, Beijing, China

^2^Department of Nephrology, First Medical Center of Chinese PLA General Hospital, State Key Laboratory of Kidney Diseases, National Clinical Research Center for Kidney Diseases, Beijing Key Laboratory of Medical Devices and Integrated Traditional Chinese and Western Drug Development for Severe Kidney Diseases, Beijing Key Laboratory of Digital Intelligent TCM for the Prevention and Treatment of Pan-vascular Diseases, Key Disciplines of National Administration of Traditional Chinese Medicine(zyyzdxk-2023310), Innovation Team and Talents Cultivation Program of National Administration of Traditional Chinese Medicine. (No: ZYYCXTD-D-202402), Beijing, China

^3^Beijing Engineering Research Center for Experimental Animal Models of Human Diseases, Institute of Laboratory Animal Science, Peking Union Medicine College, Chinese Academy of Medical Sciences, Beijing, China.

**^#^**These authors contributed to the work equally and should be regarded as **co-first authors.**

^*^**Corresponding Author:** Yuqiong Zhao*,* Email: [zhaoyuqiong@163.com](mailto:zhaoyuqiong@163.com); Tao Jiang, Email: [laoai2915@163.com](mailto:laoai2915@163.com). Medical Innovation Research Department, Chinese PLA General Hospital, Beijing, China.

**Supplementary Data:**


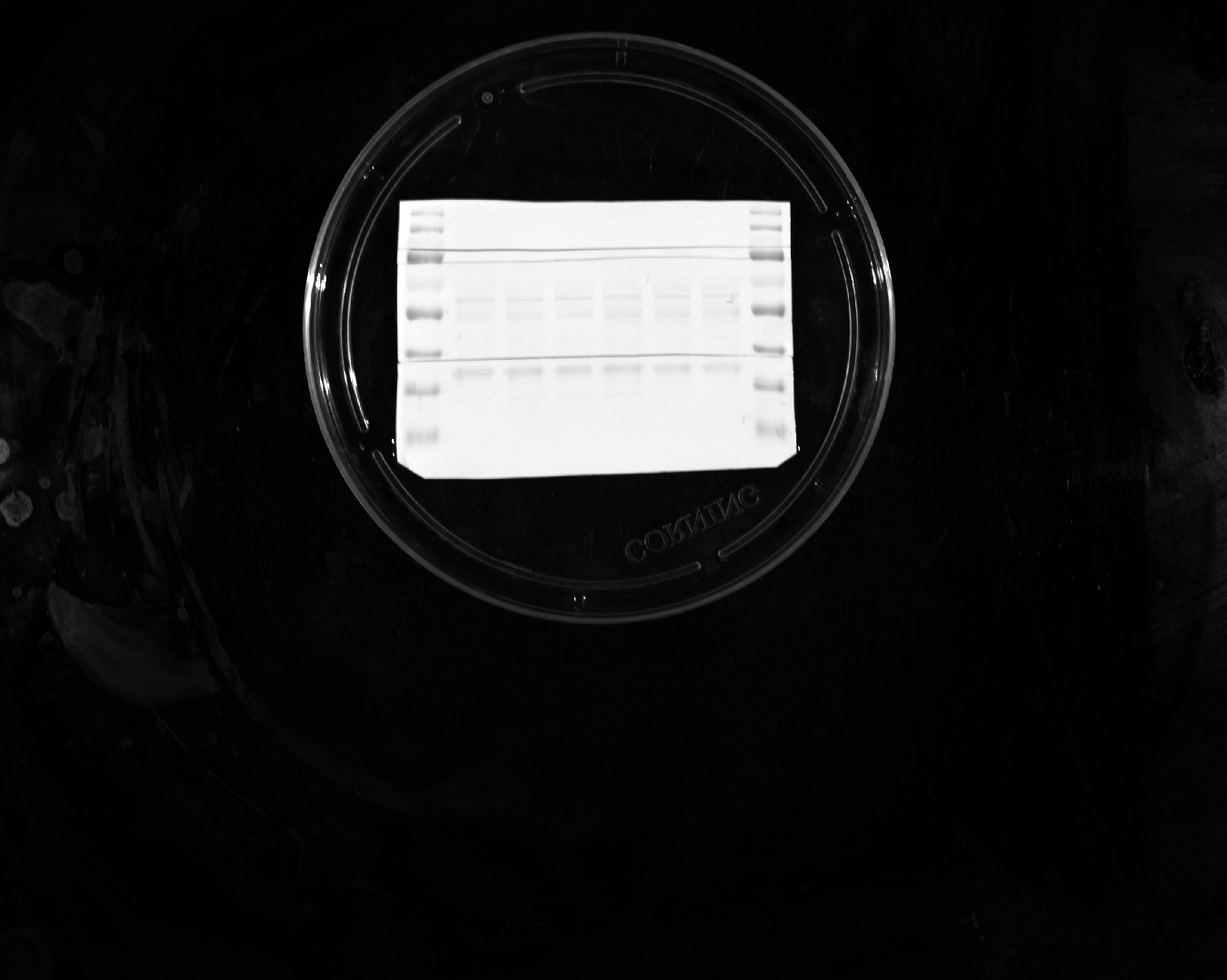

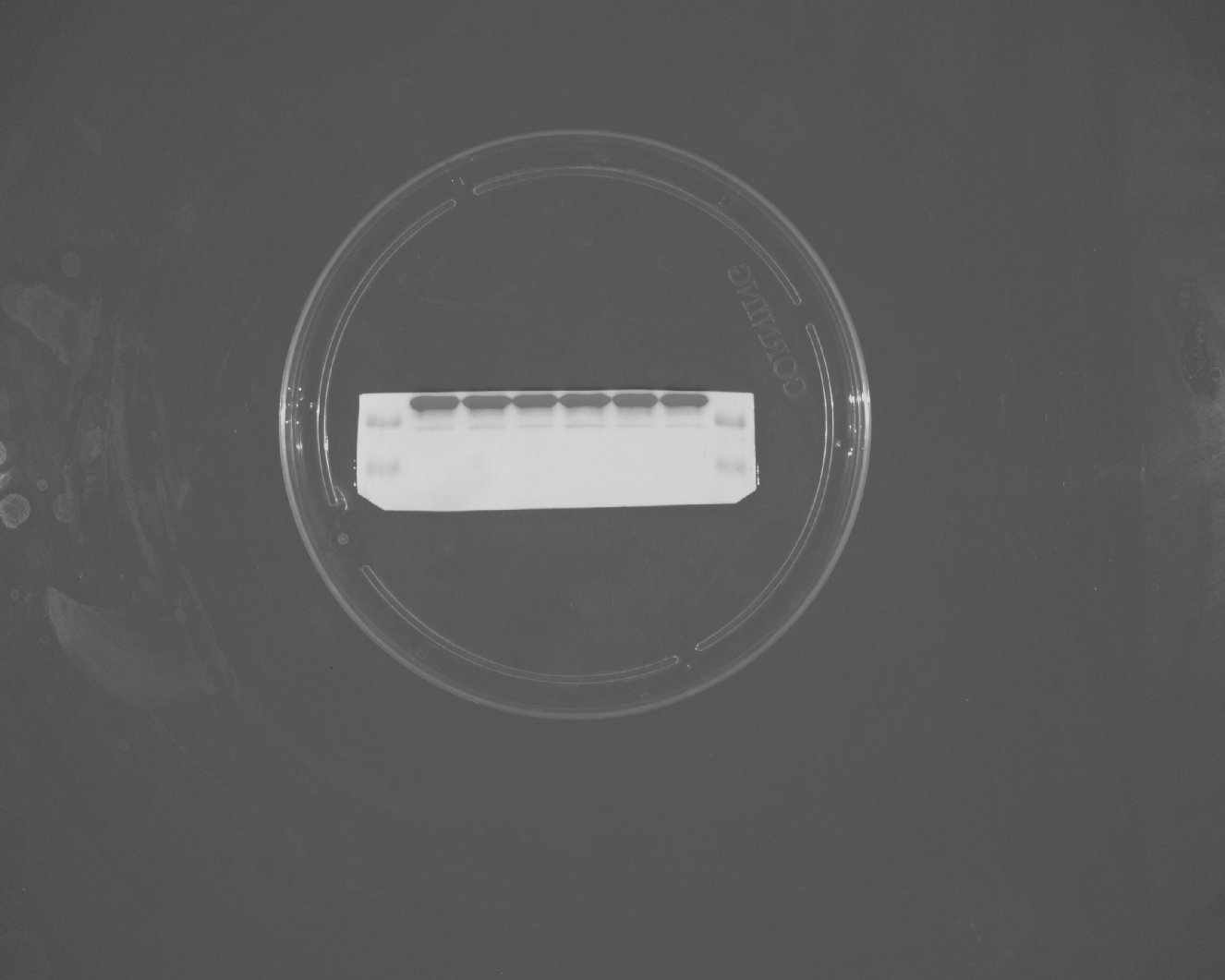

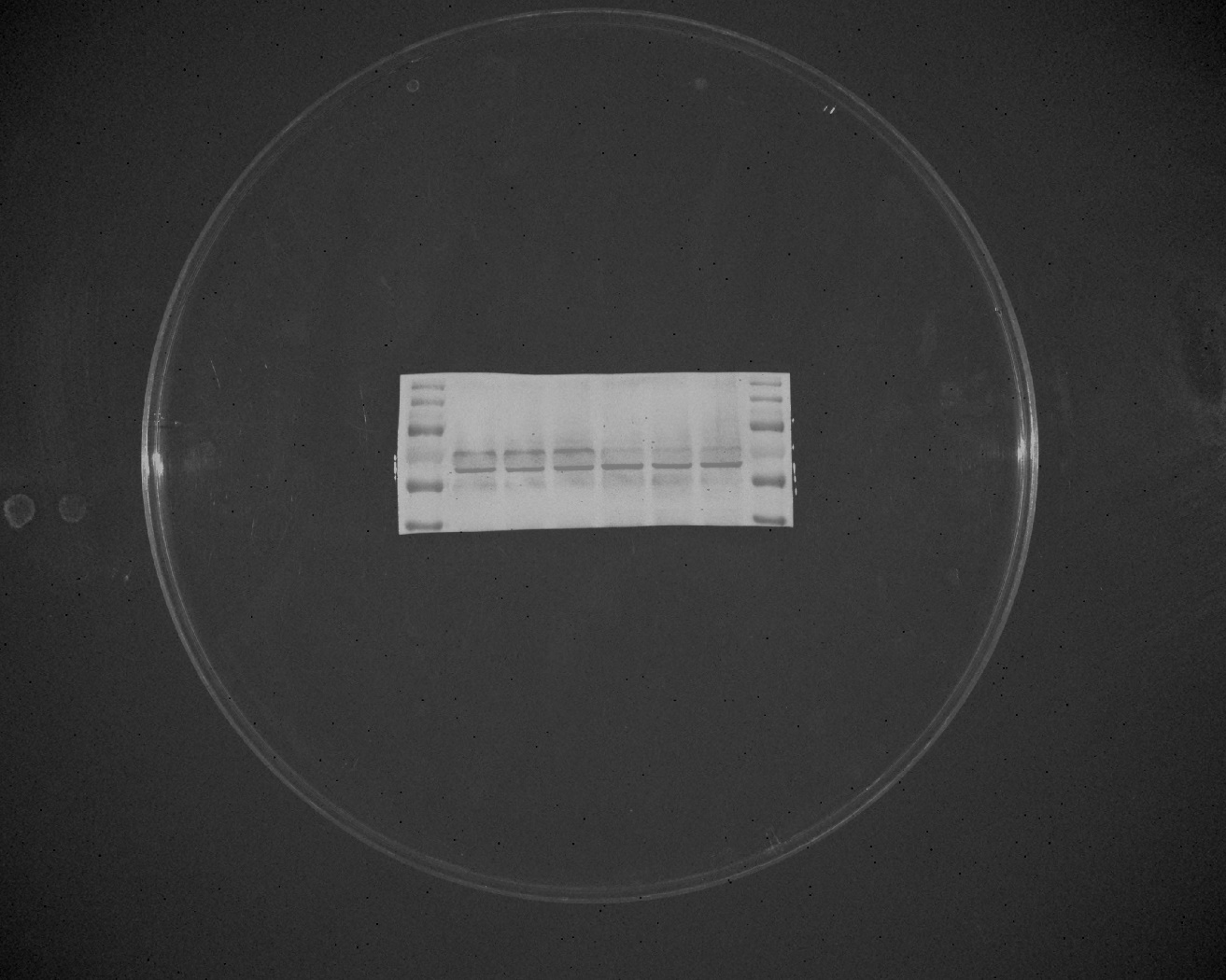

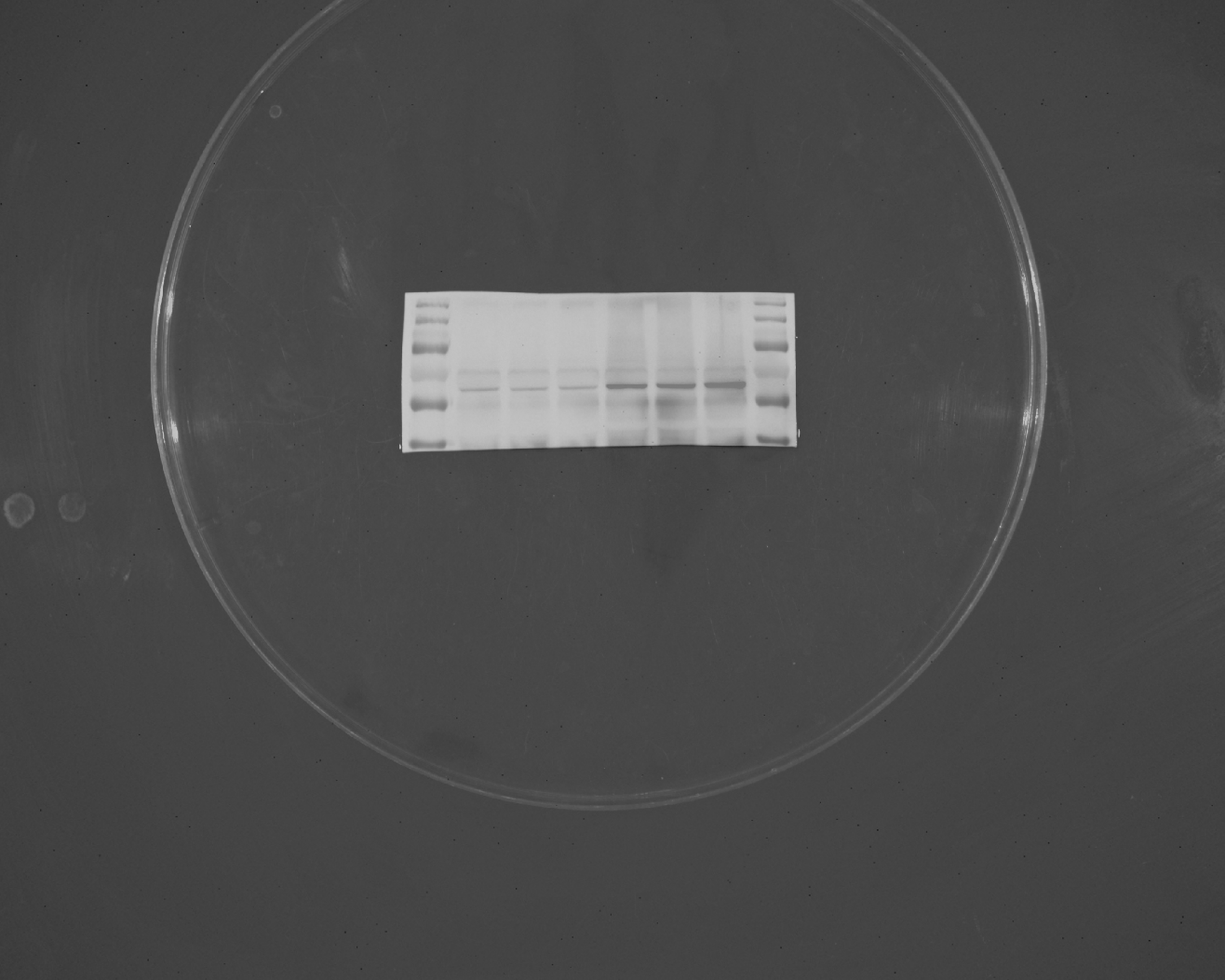

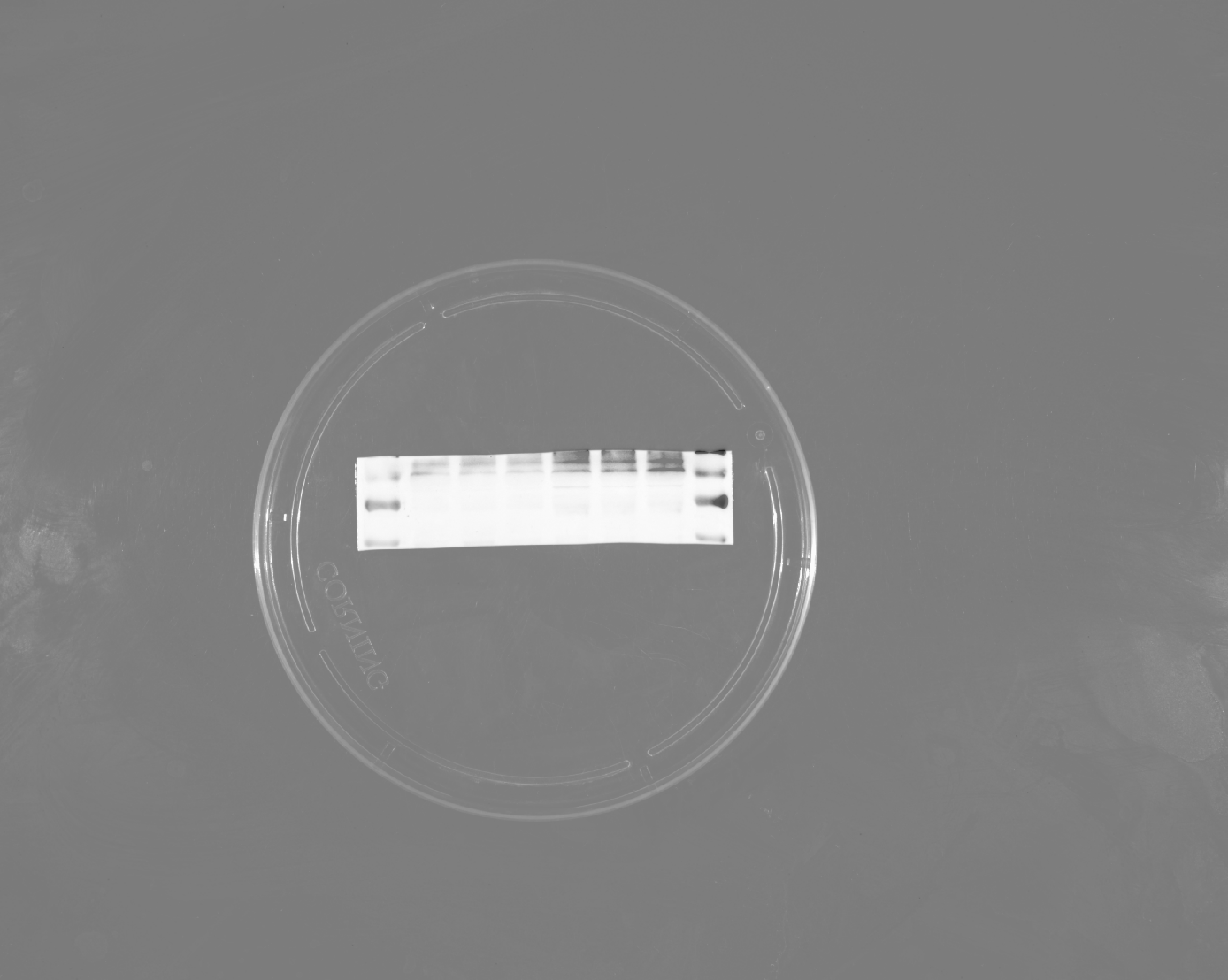

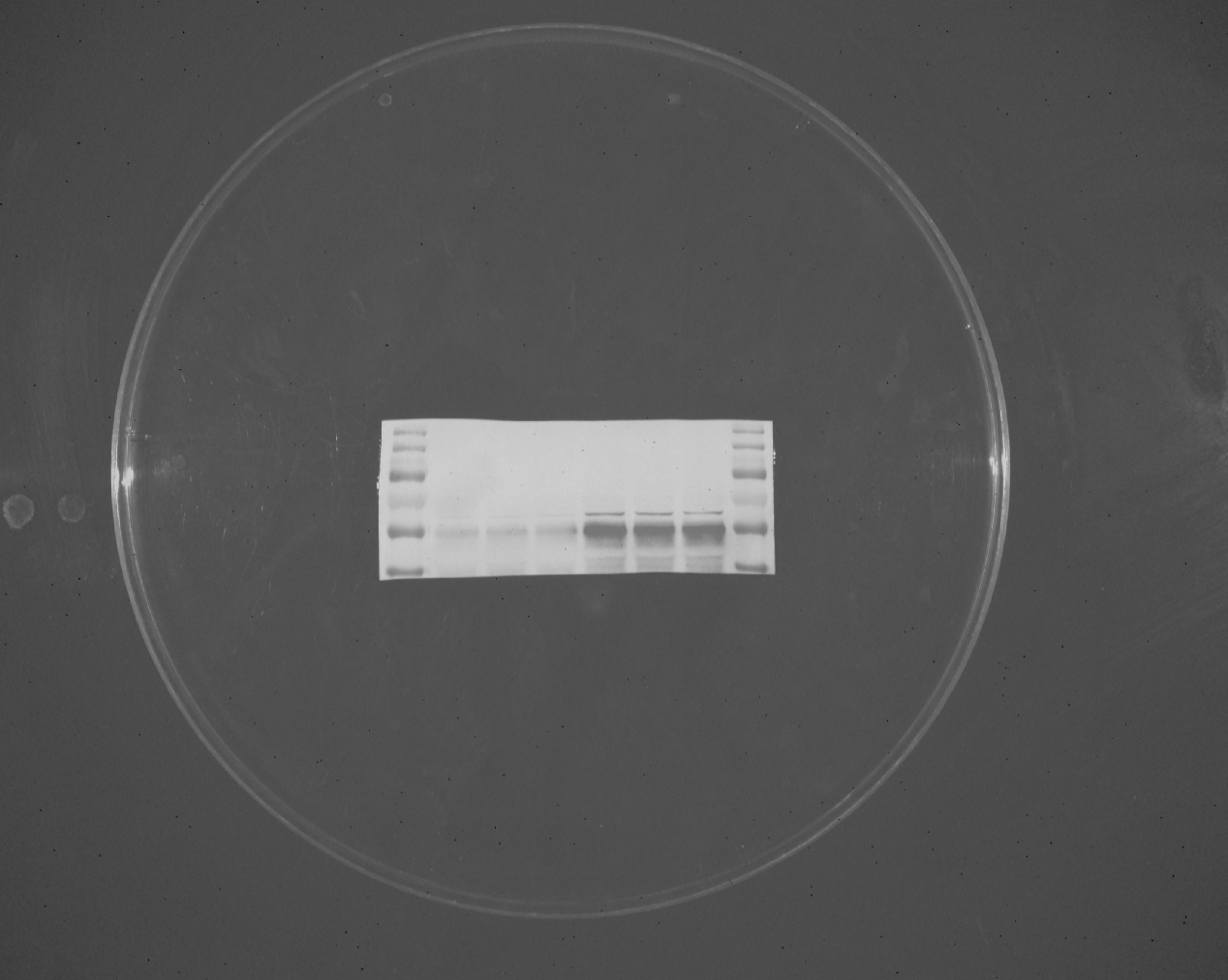
According to the image integrity and standard requirements of AMEM, we provide full length gels and blot.

170KDa -

130KDa -

100KDa -

70KDa -

55KDa -

40KDa -

35KDa -

25KDa -

- 36KDa GAPDH

25KDa -

35KDa -

170KDa -

70KDa -

55KDa -

130KDa -

100KDa -

40KDa -

- 60KDa t-AKT

- 60KDa p-AKT

130KDa -

170KDa -

100KDa -

70KDa -

55KDa -

40KDa -

- 85KDa LRP1

70KDa -

55KDa -

40KDa -

100KDa -

170KDa -

130KDa -

- 53KDa CLU

70KDa -

40KDa -

55KDa -
